# Gender-based distributional skewness of the United Republic of Tanzania’s health workforce cadres: a cross-sectional health facility survey

**DOI:** 10.1186/1478-4491-11-28

**Published:** 2013-06-24

**Authors:** Amon Exavery, Angelina M Lutambi, Neema Wilson, Godfrey M Mubyazi, Senga Pemba, Godfrey Mbaruku

**Affiliations:** 1 Ifakara Health Institute, Plot 463, Kiko Avenue, off Mwai Kibaki Road, P.O. Box 78373, Mikocheni, Dar es Salaam, Tanzania; 2 National Institute for Medical Research (NIMR), P.O. Box 9653, Dar es Salaam, Tanzania; 3 Tanzanian Training Centre for International Health (TTCIH), Mlabani Passage, P.O. BOX 39, Ifakara, Tanzania

**Keywords:** Health workforce, Gender, Distribution, Skewness, Tanzania

## Abstract

**Background:**

While severe shortages, inadequate skills and a geographical imbalance of health personnel have been consistently documented over the years as long term critical challenges in the health sector of the United Republic of Tanzania, there is limited evidence on the gender-based distribution of the health workforce and its likely implications. Extant evidence shows that some people may not seek healthcare unless they have access to a provider of their gender. This paper, therefore, assesses the gender-based distribution of the United Republic of Tanzania’s health workforce cadres.

**Methods:**

This is a secondary analysis of data collected in a cross-sectional health facility survey on health system strengthening in the United Republic of Tanzania in 2008. During the survey, 88 health facilities, selected randomly from 8 regions, yielded 815 health workers (HWs) eligible for the current analysis. While Chi-square was used for testing associations in the bivariate analysis, multivariate analysis was conducted using logistic regression to assess the relationship between gender and each of the cadres involved in the analysis.

**Results:**

The mean age of the HWs was 39.7, ranging from 15 to 63 years. Overall, 75% of the HWs were women. The proportion of women among maternal and child health aides or medical attendants (MCHA/MA), nurses and midwives was 86%, 86% and 91%, respectively, while their proportion among clinical officers (COs) and medical doctors (MDs) was 28% and 21%, respectively. Multivariate analysis revealed that the odds ratio (OR) and 95% confidence interval (CI) that a HW was a female (baseline category is “male”) for each cadre was: MCHA/MA, OR = 3.70, 95% CI 2.16-6.33; nurse, OR = 5.61, 95% CI 3.22-9.78; midwife, OR = 2.74, 95% CI 1.44-5.20; CO, OR = 0.08, 95% CI 0.04-0.17 and MD, OR = 0.04, 95% CI 0.02-0.09.

**Conclusion:**

The distribution of the United Republic of Tanzania’s health cadres is dramatically gender-skewed, a reflection of gender inequality in health career choices. MCHA/MA, nursing and midwifery cadres are large and female-dominant, whereas COs and MDs are fewer in absolute numbers and male-dominant. While a need for more staff is necessary for an effective delivery of quality health services, adequate representation of women in highly trained cadres is imperative to enhance responses to some gender-specific roles and needs.

## Background

Skewed distribution of the health workforce is a global phenomenon [1] that intensifies the well-known crisis of scarce personnel in many countries’ health sectors. This greatly affects low- and middle-income countries (LMIC), although sub-Saharan Africa (SSA) remains the most affected region [2,3]. The shortage of health personnel in absolute numbers which has received a considerable attention among researchers is one of the health systems’ pressing problems in developing countries [4,5] including Tanzania [6]. This has been acknowledged as a serious threat towards meeting the Millennium Development Goals (MDG) [7-9]. According to Barden-O’Fallon *et al*. [10], accurate knowledge of characteristics of the health workforce that affect health care production is of critical importance to health planners and policymakers. A global picture shows that skewed distribution of the health workforce geographically, and by specific characteristics, poses significant challenges to quality health care delivery [3]. Some forms of the skewness include skill mix, over specialization and gender [1], the latter being subtly discussed. So far, gender has been much less considered in health workforce-related matters both in the formal and informal systems of health care production [11,12]. However, a clear gender component reportedly exists in several ways. In formal health care systems, for example, women are less likely to be in senior, managerial and policy making roles than their male counterparts [13,14], whereas non-institutional care for the sick is often carried out by women [15]. Since poor health care utilization for some individuals due to the absence of a provider of a particular gender has been reported [16], gender balance in the cadres of health care providers may be an imperative response.

The United Republic of Tanzania’s health workforce is reportedly small, both by international and national standards [2]. In 2006, the Ministry of Health and Social Welfare (MoHSW) estimated a massive shortage in the health workforce by 65% of the staffing requirement [17]. This situation was declared a threat to effective delivery of quality health services [18]. The shortage is aggravated by, among other factors, population expansion and attrition as well as increasing disease burden due to HIV/AIDS, tuberculosis (TB) and malaria [19]. It is noted that the health workforce challenges in the country are significantly related to poor working conditions, a situation that drives some staff, especially highly trained ones, to seek employment outside the country (brain drain); those who remain working in the country become greatly demoralized [20]. A cross-sectional study conducted by Leshabari and colleagues [21] at the Muhimbili National Hospital (MNH) to assess health workers’ service and care delivery motivation found that nearly 50% of doctors and nurses were not satisfied with their jobs. Low salaries, persistent unavailability of the necessary equipment for service delivery, inadequate performance evaluation and feedback, poor communication between workers and management and lack of participation in decision-making were among major reasons reported for the dissatisfaction.

Moreover, previous evaluations have established that the health workforce in the United Republic of Tanzania is inequitably distributed [3]. There are more health personnel in urban-based health facilities than their rural counterparts [1]. xEven between districts, disparities in staffing levels exist. This is partly due to the fact that some districts host regional or tertiary hospitals, thus requiring more health personnel than the ordinary district hospitals [3]. The problem is more serious in cases where new districts were formed after dividing the original districts. There is also geographical skewness in the skill mix distribution among the existing cadres, whereby the most skilled and specialized personnel are less likely to work in rural facilities [6]. The most qualified health personnel are concentrated in a few centralized locations mainly in urban or peri-urban centres where they can access basic social services and desirable infrastructural facilities, while severe understaffing reigns at dispensary levels, especially those located in rural and peripheral settings [22].

Apart from these challenges, most of which are basically distributional, there is a general lack of empirical analysis of the status of the gender-based distribution of the health workforce in the United Republic of Tanzania. The slim evidence available shows a link between gender and geographical imbalance in the distribution of the United Republic of Tanzania’s health workforce. Reportedly, while rural facilities are severely understaffed, they are also less likely to be served by female providers [10]. Evidence from other countries shows the presence of more women in lower-status health occupations usually performed by personnel of low education on the one hand and fewer women than men among highly trained professional staff for direct health service delivery and management positions on the other [23]. The distributional skewness favours women among nurses, but women are poorly represented among doctors, dentists, pharmacists, clinical officers and managers [23]. Poor representation of women in higher-status health cadres may lead to poorer understanding of problems that are specific to women [24]. It has been further reported that female general practitioners practice differently from their male counterparts. They are capable of managing a variety of medical conditions with some differences due to patient mix and patient selectivity. On the other hand, research studies reveal that some women in more traditional areas will not seek health care for themselves or even for their children unless they have access to a female provider [25]. It has also been established that women cannot be seen by male doctors in some parts of the world [16].

While gender imbalance in the distribution of the health workforce may be affecting service production, delivery and utilization in the United Republic of Tanzania, there is no evidence available to reveal the status of gender in the distribution of HWs. The gender-based distribution of the health workforce remains barely addressed, and no literature so far clearly documents the situation in the United Republic of Tanzania. Even the few attempts in other countries that have discussed gender in relation to the distribution of the health workforce, lack details as they did not focus solely on gender. As a result, they have simply reported frequencies, without considering such robust techniques of analysis as regressions, where gender skewness could be assessed while other characteristics, such as location, age, education and so on, were controlled for. This paper, thus, has two objectives: (1) to examine the composition of the United Republic of Tanzania’s health workforce cadres by gender and (2) to assess the predictive effect of gender on each of the United Republic of Tanzanian health cadres surveyed, namely, maternal and child health aide or medical attendant (MCHA/MA), nurse, midwife, clinical officer (CO) and medical doctor (MD), using multivariate logistic regression.

## Methods

### Study design, sampling, area and population

This is a secondary analysis of data collected in 2008 in a cross-sectional health facility survey in the United Republic of Tanzania. The survey was conducted by the Ifakara Health Institute (IHI), United Republic of Tanzania, in collaboration with Columbia University, USA, as part of the implementation of the Health Systems Strengthening for Equity (HSSE) project. Based on the eight United Republic of Tanzanian zones, one region from each zone was selected randomly through a multi-stage sampling technique which brought up eight regions: Dodoma, Pwani, Mwanza, Tanga, Mbeya, Iringa, Tabora and Mtwara. From each district in these regions, two health facilities (one hospital and/or one health centre) providing emergency obstetric care (EmOC) were selected. This made a total of 88 health facilities from which 825 health workers (HWs) participated in the survey. Of these HWs, 815 (98.8%) with non-missing data on gender and cadre were extracted from the parent database for the current analysis.

### Data collection tool

Selected HWs for the primary study responded to a self-administered provider questionnaire which comprised mostly closed-ended questions and a few open-ended ones. Broadly, the questions pertained to the HWs’ background and employment, pre- or in-service training programs attended, feelings of job satisfaction, and a discrete choice experiment which aimed at understanding factors that affect employment preferences. Following interviewer training, the tool was pre-tested in facilities similar to those actually surveyed to check for relevance and answerability of the survey questions.

### Data management and statistical analyses

Original cadres were operationally regrouped by merging those that were closely related because the numbers of respondents for some cadres, such as specialists, were very small. Maternal and child health aide (MCHA), medical attendant (MA) and nursing assistant were joined to form a single category, “MCHA/MA”; registered public health nurse (PHN), enrolled public health nurse (EPHN), registered nurse (RN) and enrolled nurse (EN) were combined into a single category and referred to as “nurse”; registered midwife and enrolled midwife were grouped together as “midwife”; clinical officer (CO) remained unchanged; and assistant medical officer (AMO), medical officer (MO) and specialist were combined and referred to as “medical doctor (MD)”.

Gender-specific proportions of the HW in various categories of socio-demographic characteristics were calculated. The degree of association between gender and sociodemographic characteristics was tested using Pearson’s Chi-square (*χ*^2^) and Student’s t-tests for categorical and continuous variables, respectively. Further analyses were performed using multivariate logistic regression to assess the relationship between gender and each of the cadres, controlling for potential confounders. Each of these cadres was assessed as a separate dependent variable with two (binary) categories that classified a HW as either an MD or not an MD, CO or not a CO and so on. As coding of the outcome variable in logistic regression requires, a code of ‘1’ was assigned if a HW belonged to a particular cadre and ‘0’ if not. Therefore, the probability that a HW was an MD for example was expressed in a multivariate logistic regression model as:

$$\hat{p}=\frac{e^{\left(b_{0}+b_{1}X_{1}+b_{2}X_{2}+\ldots +b_{k}X_{k}\right)}}{1+e^{\left(b_{0}+b_{1}X_{1}+b_{2}X_{2}+\ldots +b_{k}X_{k}\right)}}$$

where $\hat{p}$ is the expected probability that a HW is an MD; X_1_ through X_k_ are k distinct independent variables; and b_0_ through b_k_ are the regression coefficients. The model was then re-written with the outcome expressed as the expected natural logarithm of the odds that a HW is an MD as:

$$\mathrm{In}\;\left(\frac{\hat{p}}{1-\hat{p}}\right)=b_{0}+b_{1}X_{1}+b_{2}X_{2}+\ldots +b_{k}X_{k}$$

The main independent variable was gender (female = 1, male = 0). This variable was taken along with other several independent variables including age, educational attainment, region, health facility ownership, and health facility type. Educational attainment was included because of evidence from other countries showing that some women do not prefer courses that take a long time to graduate, resulting in fewer women in specialized professional jobs [26]. Facility type was included in the analysis as an indicator of facility location. In the United Republic of Tanzanian health system context, a hospital is the highest level of care that serves either a region (regional hospital) or a district (district hospital) and is usually located in the headquarters of regions or districts. Therefore, it may be appropriate to consider hospital locations in the United Republic of Tanzania as urban. Health centres on the other hand, which are the second highest level of care in the United Republic of Tanzania, exist mostly in rural and sometimes in urban settings. Therefore, this variable to a larger extent reflects the rural–urban distribution of HWs in the country. Data analysis was performed using STATA (Version 11) statistical software (Stata Corp, Texas, USA).

### Ethical approval

Ethical approval to conduct the main survey from which this paper stems was granted by the Medical Research Coordinating Committee (MRCC) of the National Institute for Medical Research (NIMR) in the United Republic of Tanzania. Participation in the study was voluntary with all consenting individuals having to sign an informed consent form first. To ensure integrity and confidentiality, the database was anonymous with no information (e.g. names) that could identify the participant. Storage of completed questionnaires and consent forms was carefully managed, and access to the data was restricted to a few experts.

## Results

**Figure 1:**
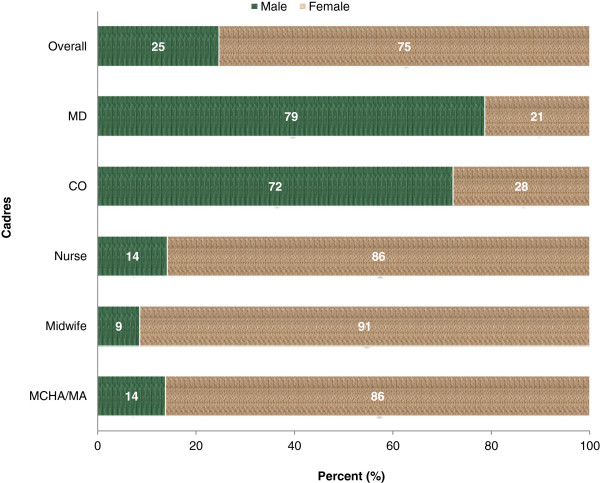
Composition of the United Republic of Tanzania’s health workforce cadres by gender (n = 815), 2008.

Of the 815 HWs analyzed, 75% were women. Their mean age was 39.7 ± 9.0 years, ranging from 15 to 63. The proportions of female staff in different cadres were as follows: MCHA/MA, 86%; nurse, 86%; midwife, 91%; CO, 28%; and MD, 21% (Figure 1).

Table 1 presents the distribution of female and male HWs by their background characteristics. The contribution of each cadre to the total number of HWs was: MCHA/MA, 21.4%; nurse, 26.9%; midwife, 31.5%; CO, 11.0%; and MD, 9.2%. These proportions were gender maldistributional (*P* < 0.001), with female HWs being the majority among MCHA/MA, nurses and midwives but the minority among COs and MDs. With respect to the highest level of education attained, 59.6% (70.2% female and 27.4% male) were certificate holders; 28.7% (24.2% female and 42.1% male) held an ordinary diploma while 11.8% (5.6% female and 30.5% male) held at least an advanced diploma. These differences in the highest level of education by gender were statistically significant (*P* < 0.001).

**Table 1:** Gender-bases distribution of the United Republic of Tanzania’s health workforce by background characteristics, 2008 (n = 815)

**Characteristic**			**Gender**			**Total**	***P*-Value**
		**Male**		**Female**			
	**n**	**%**	**n**	**%**	**n**	**%**	
**Professional background (cadres)**	**201**	**100.0**	**614**	**100.0**	**815**	**100.0**	<0.001
MCHA/MA	24	11.9	150	24.4	174	21.4	
Nurse	31	15.4	188	30.6	219	26.9	
Midwife	22	11.0	235	38.3	257	31.5	
CO	65	32.3	25	4.1	90	11.0	
MD	59	29.4	16	2.6	75	9.2	
**Age group(years)**	**189**	**100.0**	**580**	**100.0**	**769**	**100.0**	<0.001
15-24	4	2.1	14	2.4	18	2.3	
25-34	36	19.1	215	37.1	251	32.6	
35-44	72	38.1	184	31.7	256	33.3	
45-54	58	30.7	127	21.9	185	24.1	
55-64	19	10.1	40	6.9	59	7.7	
Mean (Male = 42.2 ± 9.3; Female = 38.9 ± 9.4)						.	
**Region**	**201**	**100.0**	**614**	**100**	**815**	**100**	0.002
Dodoma	28	13.9	85	13.8	113	13.9	
Iringa	21	10.5	95	15.5	116	14.2	
Mbeya	29	14.4	102	16.6	131	16.1	
Mtwara	16	8.0	23	3.8	39	4.8	
Mwanza	24	11.9	85	13.8	109	13.4	
Pwani	30	14.9	71	11.6	101	12.4	
Tabora	37	18.4	65	10.6	102	.12.5	
Tanga	16	8.0	88	14.3	104	12.8	
**Highest level of education completed**	**190**	**100.0**	**574**	**100.0**	**764**	**100.0**	<0.001
Certificate	52	27.4	403	70.2	455	59.6	
Diploma	80	42.1	139	24.2	219	28.7	
Advanced diploma/higher	58	30.5	32	5.6	90	11.8	
**Leaving level for basic education**	**199**	**100.0**	**608**	**100.0**	**807**	**100.0**	<0.001
Primary	36	18.1	167	27.5	203	25.2	
Ordinary secondary	129	64.8	403	66.3	532	65.9	
Advanced secondary	29	14.6	18	3.0	47	5.8	
Others	5	2.5	20	3.3	25	3.1	
**Facility ownership**	**201**	**100.0**	**614**	**100.0**	**815**	**100.0**	0.995
Government	181	90.1	553	90.1	734	90.1	
Private	20	9.9	61	9.9	81	9.9	
**Facility type**	**201**	**100.0**	**614**	**100.0**	**815**	**100.0**	0.125
Health centre	77	38.3	199	32.4	276	33.9	
Hospital	124	61.7	415	67.6	539	66.1	

*MCHA* maternal and child health aide, *MA* medical attendant, *CO* clinical officer, *MD* medical doctor.

With regard to the regions where the HWs worked, 13.9% (13.8% female and 13.9% male) worked in Dodoma; 14.2% (15.5% female and 10.5% male) worked in Iringa, 16.1% (16.6% female and 14.4% male) worked in Mbeya; 4.8% (3.8% female and 8.0% male) worked in Mtwara. Also, 13.4% (13.8% female and 11.9% male) worked in Mwanza, 12.4% (11.6% female and 14.9% male) worked in Pwani; 12.5% (10.6% female and 18.4% male) worked in Tabora and 12.8% (14.3% female and 8.0% male) worked in Tanga. Gender differences across the regions were statistically significant (*P* = 0.002). Although the distribution of the female and male HWs by health facility ownership was symmetric (*P* = 0.995), about 90% of the HWs worked in government facilities. Also, about two-thirds (66.1%) of all HWs worked in hospitals and the rest in health centres with no significant differences by gender (*P* = 0.125).

### Regression results

The relationship between gender and the cadres of the United Republic of Tanzania’s health workforce was further assessed in a multivariable style, adjusting for age, education, region, health facility ownership and health facility type (Table 2). The results reveal a massive distributional skewness of the workforce by gender. The odds of an MCHA/MA being a woman was 3.70 times higher than that for their male counterparts (OR = 3.70, 95% CI 2.16‒6.63). Similarly, a nurse was 5.61 times more likely to be a woman than to be a man (OR = 5.61, 95% CI 3.22‒9.78). It was also almost three times more likely that a midwife was a woman (OR = 2.74, 95% CI 1.44‒5.20). On the other hand, the chance that a CO was a woman was 92% less likely than being a man (OR = 0.08, 95% CI 0.04‒0.17) and, likewise, an MD was 96% less likely to be a woman than to be a man (OR = 0.04, 95% CI 0.02‒0.09).

**Table 2:** Multivariate logistic regression models of the effect of gender on cadres of the United Republic of Tanzania’s health workforce, 2008

	**Cadres**									
	**MCHA/MA** **versus** **not CHA/MA** **(n = 769)**		**Nurse** **versus** **not Nurse** **(n = 725)**		**Midwife** **versus** **not Midwife** **(n = 725)**		**CO** **versus** **not CO** **(n = 725)**		**MD** **versus** **not MD** **(n = 769)**	
**Variable**	**Odds ratio (OR)**	**95% confidence interval (CI)**	**Odds ratio (OR)**	**95% confidence interval (CI)**	**Odds ratio (OR)**	**95% confidence interval (CI)**	**Odds ratio (OR)**	**95% confidence interval (CI)**	**Odds ratio (OR)**	**95% confidence interval (CI)**
**Gender**										
Male	1.00	--	1.00	--	1.00	--	1.00	--	1.00	--
Female	3.70***	2.16-6.33	5.61***	3.22-9.78	2.74***	1.44-5.20	0.08***	0.04-0.17	0.04***	0.02-0.09
**Age (in years)**	1.06***	1.04-1.08	1.02*	1.00-1.04	0.93***	0.91-0.96	1.00	0.96-1.04	1.04**	1.01-1.07
**Highest level for education completed**										
Certificate	--	--	1.00	--	1.00	--	1.00	--	--	--
Diploma	--	--	15.07***	9.45-24.03	0.02***	0.01-0.05	46.45***	16.83-128.22	--	--
Advanced diploma/higher	--	--	0.96	0.41-2.23	0.08***	0.03-0.21	0.56	0.10-3.17	--	--
**Region**										
Dodoma	1.00	--	1.00	--	1.00	--	1.00	--	1.00	--
Iringa	0.80	0.38-1.71	1.08	0.52-2.23	2.07*	0.97-4.40	0.16***	0.03-0.86	0.04***	0.01-0.36
Mbeya	1.90*	0.97-3.74	0.53	0.25-1.13	0.75	0.36-1.59	0.75	0.21-2.74	0.80	0.30-2.11
Mtwara	1.04	0.36-3.00	0.59	0.20-1.77	3.88**	1.24-12.19	3.68	0.71-19.01	0.37	0.10-1.34
Mwanza	2.47**	1.22-4.99	0.90	0.40-2.05	1.13	0.51-2.54	1.54	0.37-6.37	0.42	0.13-1.31
Pwani	1.26	0.60-2.69	0.60	0.28-1.32	0.74	0.34-1.64	3.35**	1.02-11.01	0.34**	0.12-0.97
Tabora	0.84	0.38-1.86	0.88	0.42-1.86	1.05	0.47-.2.33	2.62	0.75-9.16	0.20***	0.07-0.57
Tanga	053	0.24-1.19	2.04**	1.01-4.12	0.57	0.26-1.25	0.65	0.16-2.65	1.17	0.43-3.18
**Facility ownership**										
Government	1.00	--	1.00	--	1.00	--	1.00	--	1.00	--
Private	2.73***	1.52-4.89	1.16	0.59-2.28	0.33***	0.16-0.66	0.89	0.25-3.11	0.52	0.17-1.57
**Facility type**										
Health centre	1.00	--	1.00	--	1.00	--	1.00	--	1.00	--
Hospital	0.47***	0.32-0.71	1.28	0.83-2.00	2.72***	1.71-4.32	0.14**	0.06-0.32	3.98***	1.92-8.25

***P < 0.01, **P < 0.05, *P < 0.10; *MCHA/MA* maternal and child health aide or medical attendant, *CO* clinical officer, *MD* medical doctor.

Apart from gender, there were other factors with a significant association with the cadres. A one year increase in age was associated with a 6% increased likelihood of a health worker being an MCHA/MA (OR = 1.06, 95% CI 1.04‒1.08); 7% less likely to be a midwife (OR = 0.93, 95% CI 0.91‒0.96); and 4% more likely to be an MD (OR = 1.04, 95% CI 1.01‒1.07). In terms of educational attainment, nurses were almost 15 times more likely to be holding an ordinary diploma than a certificate (OR = 15.07, 95% CI 9.45‒24.03), whereas midwives were 98% and 92% less likely to be holders of an ordinary diploma (OR = 0.02, 95% CI 0.01‒0.05) and advanced diploma or higher (OR = 0.08, 95% CI 0.03‒0.21), respectively, than a certificate. COs were about 46 times more likely to hold an ordinary diploma than a certificate (OR = 46.45, 95% CI 16.83‒128.22).

There was also a geographical imbalance in the distribution of some cadres. With Dodoma being a baseline or a reference category, there was a higher likelihood of more MCHA/MAs in Mwanza than Dodoma (OR = 2.47, 95% CI 1.22‒4.99). The presence of nurses in Tanga was twice as likely as in Dodoma (OR = 2.04, 94% CI 1.01‒4.12). Also, midwives were about four times more likely in Mtwara than in Dodoma (OR = 3.88, 95% CI 1.24‒12.19). While the presence of COs in Iringa was 84% less likely compared to Dodoma (OR = 0.16, 95% CI 0.03‒0.86), it was 3.35 times more likely that they were present in Pwani than in Dodoma (OR = 3.35, 95% CI 1.02‒11.01). Furthermore, the availability of MDs was 96% less likely in Iringa (OR = 0.04, 95% CI 0.01‒0.36), 66% less likely in Pwani (OR = 0.34, 95% CI 0.12‒0.97) and 80% less likely in Tabora (OR = 0.20, 95% CI 0.07‒0.57) compared to the Dodoma region.

With respect to health facility ownership, MCHA/MAs were about three times more likely to be working in private facilities than in government facilities (OR = 2.73, 95% CI 1.52‒4.89), whereas midwives were 67% less likely to be working in private facilities compared to government facilities (OR = 0.33, 95% CI 0.16‒0.66). In terms of facility type, MCHA/MAs were 53% less likely to be working in hospitals than health centres. Similarly, COs were 86% less likely to be working in hospitals than health centres. In contrast, midwives and MDs were 2.72 (OR = 2.72, 95% CI 1.71‒4.32) and 3.98 (OR = 3.98, 95% CI 1.92‒8.25) times more likely to be working in hospitals than in health centres, respectively.

## Discussion

This paper sought to examine the gender-based distribution of the United Republic of Tanzania’s health workforce cadres. This question has so far received limited attention in previous analyses [11,12]. Our findings demonstrate that the distribution of the health cadres in the United Republic of Tanzania is dramatically gender skewed, probably reflecting gender inequality in health career choices. Nursing and midwifery cadres are generally large and female-dominant, while CO and MD cadres are small in absolute numbers and male-dominant. The possibility of this happening may be associated with cultural, familial or systemic structures underlying values and specialty preferences in choosing careers. Our findings are consistent with evidence from other studies in which female health personnel are the minority among the highly trained cadres [23], but the majority in other health specializations [27]. The overall picture of three-quarters of the HWs being women in this study area is similar to that reported in Congo where women are the majority [28].

The gender-based distributional skewness in the United Republic of Tanzanian health workforce cadres persisted and remained highly significant even after controlling for other variables in a multivariate logistic regression model. There is a possibility that care-related jobs, such as nursing and midwifery, attract more women than men in the United Republic of Tanzania. There is also a chance that the services delivered by people from the MCHA/MA, nursing and midwifery cadres tend to be perceived as more suitable for women than men. This, however, needs further elucidation. Previous studies have shown that the dominance of women among nurses is a historical phenomenon [29] and generally apparent in lower cadres [30]. That is why it is important to examine why this has been so and whether achieving a balance can in any way reduce the HW shortage.

We observed the presence of more female- than male-holders of certificates, while the proportion of women declined rapidly among those with higher qualifications. Although the higher the qualification the lower was the proportion of both women and men, the data show that the proportion of men outnumbered their female counterparts for diploma, advanced diploma and higher qualifications. This trend somewhat prevailed in terms of the leaving level for basic education, such that the proportion of women with primary education was 1.5 times as high as that for men, whereas the proportion of those holding an ordinary secondary education was similar between women and men. However, the proportion of women with advanced secondary education which is a key entry point into colleges and universities for higher education was five times as small as that of men. Leon and Kolstad [31] report a lack of primary interest in medicine among medical school entrants. The authors further report skewed recruitment and the absence of a rural-related clinical curriculum as facilitators of inequitable distribution of doctors in the United Republic of Tanzania. Although the study did not clarify the observed effect by gender, it may be that the overall effect of these factors is gender-sensitive. Further research is, therefore, needed to provide explanations.

Moreover, the demand for more specialized cadres such as MDs may be higher for hospitals than health centres because the former is a higher level of care with more specialized services than the latter. Hospitals also constitute the receiving end of the referral pathway in the health service delivery framework of the United Republic of Tanzania. That is why facility type was a significant explanatory variable of the cadres’ distribution, with hospitals being more likely than health centres to have MDs, for example. Additionally, most hospitals are in urban settings where MDs prefer to work as already noted [22]. Our findings also indicate that MCHA/MAs and COs are less likely to work in hospitals, implying somewhat that these are the likely cadres in health centres and most rural facilities where MDs in the United Republic of Tanzania are rarely present.

Generally, the gender skewness observed in this study may be linked to gender differences in entering and leaving various academic junctures. In a study on gender differences in performance in science subjects in the United Republic of Tanzania, Mushi [32] found that more girls than boys fail in science subjects in the Advanced Certificate of Secondary Education Examination (ACSEE). Also, very few girls enroll in science subjects at the universities, implying that fewer women eventually proceed to highly trained medical cadres.

We agree with previous authors that a strong health system requires among other things a strong, adequate and balanced workforce to ensure effective and efficient delivery of quality health services [17]. Therefore, we additionally emphasize that that gender should be considered important in human resources planning to ensure a balanced health workforce. This is because of the existing evidence showing that many women prefer female providers in certain kinds of health services such as those involving intimate physical examination [33,34]. Moreover, it has been reported that some people, especially women, fail to seek health care when they have no access to a provider of their gender [25]. While all this may be true in the United Republic of Tanzania, there currently exists no evidence showing whether or not this is so; thus, there is a need for researchers to provide clarifications. In this case, it may be good to explore whether or not men would prefer particular health services for their wives, such as those associated with pregnancy and childbirth, to be delivered by female or male providers. Therefore, it is important that the United Republic of Tanzanian school admission policies, recruitment and on-the-job training modalities are re-examined in order to ensure a good balance between female and male HWs, especially in highly trained cadres. This will not only respond to the shortage of HWs in the United Republic of Tanzania where records show a very low doctor-to-population ratio [35], but also cater for gender-specific roles and needs.

### Limitations and strengths

Although the present paper is limited to HWs in health facilities providing EmOC, the findings and their interpretation respond to the existing gender-related gaps in the health workforce distribution in the United Republic of Tanzania. Factors such as marital status and spouse’s place of work which may also influence one’s choice of career specialization were not available. Future research should examine more cadres at all levels of the United Republic of Tanzanian health care delivery framework.

Despite these limitations, this study makes an important contribution to the human resources for health literature by deeply considering the gender dimension in the distribution of the health workforce in the United Republic of Tanzania. Unlike some studies in the field [6,16], our study goes beyond descriptive analysis to apply multivariate analysis to control for confounding factors and see clearly the role of gender in the distribution of HWs in the United Republic of Tanzania. Gender is an aspect often overlooked in medical and general public health studies that address questions related to human resources for health in various countries [26].

## Conclusions

The gender-based distribution of the United Republic of Tanzania’s health workforce cadres is significantly skewed, with MCHA/MA, nursing and midwifery cadres being large and female-dominant, whereas CO and MD cadres are slim in absolute numbers and male-dominant. It is important that gender be considered a dimension of crucial importance in human resources planning in the United Republic of Tanzania as this will not only contribute to uplifting the United Republic of Tanzania’s doctor-to-population ratio, but will also respond to possible health service users’ preferences regarding the gender of the service providers. As a result, this may enhance health seeking behaviour and health care utilization.

## Abbreviations

ACSEE: Advanced certificate of secondary education examination; CI: Confidence interval; CO: Clinical officer; EmOC: Emergency obstetric care; HSSE: Health systems strengthening for equity; HWs: Health workers; LMIC: Low- and middle-income countries; MA: Medical attendant; MCHA: Maternal and child health aide; MD: Medical doctor; MoHSW: Ministry of health and social welfare; OR: Odds ratio; SSA: sub-Saharan Africa.

## Competing interests

All authors declare that they have no competing interests.

## Authors’ contributions

AE conceptualized the problem, designed the study, participated in field data collection, performed data analysis and drafted the manuscript. AML participated in designing the study, data analysis and critical review of the manuscript. NW participated in drafting the manuscript and critical review of it. GM oversaw the study design as well as critical review of the manuscript. SP and GMM contributed to interpretations and overall review of the manuscript. All authors read and approved the final draft.
